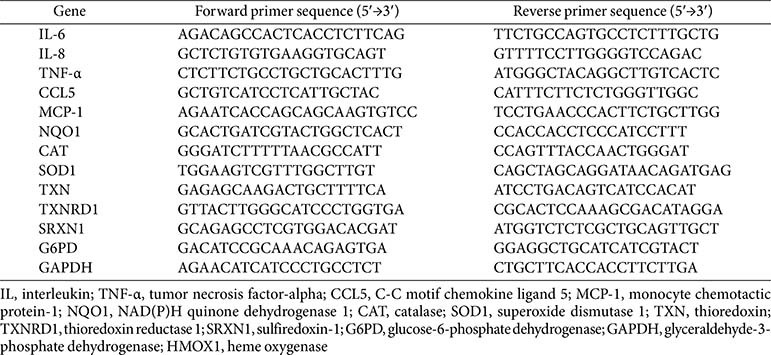# MMPP Attenuates the Inflammatory Response by Suppressing ROS and Proinflammatory Cytokine and Chemokine Production in TNF-α/IFN-γ-Stimulated Human Keratinocytes

**DOI:** 10.4014/jmb.2508.08045

**Published:** 2025-10-15

**Authors:** Chae-Min Lim, Na-Yeon Kim, Jinju Kim, Hyo-Min Park, Hee-Pom Lee, Jin-Tae Hong, Dong Oh Kwon, Kangduk Choi, Do-Young Yoon

**Affiliations:** 1 Department of Bioscience and Biotechnology, Konkuk University, Seoul 05029, Republic of Korea; 2 GIS Korea Ltd., Anseong 17513, Republic of Korea; 3 College of Pharmacy & Medical Research Center, Chungbuk National University, Cheongju 28160, Republic of Korea; 4 Department of Biotechnology, Genomic Information Center, Hankyong National University, Anseong 17579, Republic of Korea

**Keywords:** Skin inflammation, MMPP, ROS, IKK, NF-κB

## Abstract

Keratinocytes play a significant role in regulating skin immune homeostasis, and their dysregulation is central to chronic inflammatory disorders such as atopic dermatitis and psoriasis. Inflammatory mediators such as tumor necrosis factor alpha (TNF-α) and interferon-gamma (IFN-γ) can disrupt this balance, inducing abnormal cytokine and chemokine expression that promotes immune cell infiltration and chronic inflammation in the skin. While (E)-2,4-bis(p-hydroxyphenyl)-2-butenal (BHPB) has shown anti-inflammatory effects, its clinical potential is limited due to poor stability and lack of drug-like properties. To overcome these limitations, (E)-2-Methoxy-4-(3-(4-methoxyphenyl)prop-1-en-1-yl)phenol (MMPP), a stable BHPB analog, was synthesized as a potential therapeutic candidate. This study examined the antioxidant and anti-inflammatory effects of MMPP in TNF-α/IFN-γ-stimulated human HaCaT keratinocytes, assessing its impact on reactive oxygen species (ROS) production and proinflammatory signaling pathways. MMPP significantly reduced ROS levels and inhibited proinflammatory cytokine and chemokine expression by interacting with IKKα/β and suppressing the main downstream IκBα and NF-κB, with additional effects on MAPK, AP-1, and STAT1 pathways. These findings highlight the promise of MMPP as a stable, drug-like agent with therapeutic potential for skin inflammation and oxidative stress by modulating keratinocyte-mediated immune responses.

## Introduction

The skin functions as a physical barrier and immunological defender [1]. Disruption of the skin barrier in patients with atopic dermatitis or other skin diseases is attributed to abnormalities in keratinocytes, the main cell type in the epidermis of human skin [2]. Keratinocytes constitute a frontline defense by expressing diverse pattern-recognition receptors (PRRs) that detect pathogens and trigger immune responses. Upon inflammatory stimulation, they release various effector molecules such as cytokines and chemokines, which are essential for recruiting inflammatory cells [3]. For example, in response to stimulation by proinflammatory cytokines such as interleukin (IL)-6 and IL-1β, keratinocytes secrete various types of chemokines and inflammatory cytokines, such as C-C motif chemokine ligand (CCL) 17/thymus and activation-regulated chemokine (TARC), CCL22/macrophage-derived chemokine (MDC), CCL5/regulated on activation normal T-cell expression and secreted (RANTES), CCL2/monocyte chemotactic protein-1 (MCP-1), IL-8, thymic stromal lymphopoietin (TSLP), IL-25, and IL-33, which induce the infiltration of immune cells into the inflamed skin area and cause the inflammatory condition in atopic dermatitis [4-7].

Inflammatory responses have been associated with oxidative stress caused by the excessive intracellular production of reactive oxygen species (ROS). Upon their exposure to inflammatory stimuli, elevated ROS levels can overactivate transcription factors such as nuclear factor-kappa B (NF-κB) and activator protein-1 (AP-1), and mitogen-activated protein kinase (MAPK), which are the signal molecules involved in inflammatory responses. Furthermore, through activation of the inflammatory signaling pathways, the expression of inflammatory mediators such as cytokines and chemokines is increased [8]. However, nuclear factor erythroid 2-related factor 2 (NRF2)/heme oxygenase-1 (HO-1) signaling activates the expression of genes that encode antioxidants, which subsequently exert anti-inflammatory responses by mitigating ROS [9]. Organisms maintain redox homeostasis via detoxification and antioxidant enzymes such as superoxide dismutase (SOD), catalase (CAT), and NAD(P)H: quinone oxidoreductase 1 (NQO1) [10, 11].

(E)-2-Methoxy-4-(3-(4-methoxyphenyl) prop-1-en-1-yl) phenol (MMPP) is a novel synthetic compound from a library of (E)-2,4-bis(p-hydroxyphenyl)-2-butenal (BHPB) analogs. Although BHPB has been shown to exert anti-inflammatory effects, its aldehyde moiety confers poor chemical stability and suboptimal drug-like properties [12]. To overcome this, the conjugated α, β-unsaturated aldehyde moiety of BHPB was modified to yield MMPP as a more stable candidate [13]. Previous studies have shown that MMPP exerts anti-inflammatory effects in THP-1 monocytic cells stimulated with either phorbol 12-myristate 13-acetate (PMA) or lipopolysaccharide (LPS) [14, 15]. Also, MMPP was shown to inhibit H_2_O_2_ generation as well as inflammatory mediators and signaling pathways in synoviocytes derived from patients with rheumatoid arthritis [16]. These findings suggest that MMPP has therapeutic potential in attenuating inflammation and oxidative stress. However, the effects of MMPP in human HaCaT keratinocytes and the detailed mechanisms through which MMPP alleviates skin inflammation and oxidative stress, have not yet been fully elucidated. Therefore, this study aimed to investigate the antioxidant and anti-inflammatory effects of MMPP and the molecular mechanism and signaling pathways underlying those effects, using tumor necrosis factor-alpha (TNF-α)/interferon-gamma (IFN-γ)-stimulated human HaCaT keratinocytes as a cellular model [17]. Exposure of the keratinocytes to TNF-α and IFN-γ produces ROS and causes abnormal cytokine and chemokine expression, which is speculated to promote the infiltration of monocytes/T cells into the site of inflammation in the skin [18]. Particularly, we assessed the effects of MMPP on proinflammatory cytokine and chemokine expression at both gene and protein levels. Additionally, the inhibitory effects of MMPP on major molecular signaling pathways were confirmed. In this study, it was revealed the inhibitory ability of MMPP on the inhibitor of NF-κB kinase subunits alpha and beta (IKKα/β), inhibitor of NF-κB alpha (IκBα), MAPK, and transcription factors NF-κB, AP-1, and signal transducer and activator of transcription 1 (STAT1), exerting to its anti-inflammatory effects. Therefore, it could be a good alternative drug candidate as a therapeutic modulator of skin inflammation.

## Materials and Methods

### Cell Culture

The human keratinocyte HaCaT cell line was obtained from Bogoo Biological Technology (China) [19]. The cells were cultured in Dulbecco’s modified Eagle’s medium (DMEM) (Welgene Incorporation, Republic of Korea) supplemented with 10% (*v/v*) heat-inactivated fetal bovine serum (Hyclone Laboratories, USA), 100 U/ml penicillin, and 100 μg/ml streptomycin. The cells were incubated under 5% CO_2_-containing air at 37°C.

### Raw Materials and Reagents

MMPP, supplied by Professor Jin-Tae Hong of Chungbuk National University (Republic of Korea), was dissolved in dimethyl sulfoxide (DMSO) for this study. All experiments were performed with a DMSO concentration of less than 0.5% to ensure that the solvent does not affect the results, since it has no cytotoxic or anti-inflammatory effects at these low concentrations. DMSO at the same maximum concentration as that of MMPP (*i.e.*, 10 μg/ml) was also used in all experiments as a negative control. In this way, we could confirm that any observed cytotoxic or anti-inflammatory effects were solely due to MMPP and not DMSO.

### Cell Viability Assay

Cell viability was measured using the 3-(4,5-dimethylthiazol-2-yl)-5-(3-carboxymethoxy phenyl)-2-(4-sulfophenyl)-2H-tetrazolium (MTS) assay. HaCaT cells (1.5 × 10^4^ cells/well) were seeded in 96-well plates and pretreated with different concentrations of MMPP (2.5, 5, and 10 mg/ml) for 1 h, followed by stimulation with TNF-α/IFN-γ (10 ng/ml) for 24 h. The number of viable cells was estimated using the CellTiter 96 Aqueous One Solution Assay (Promega, USA) containing MTS and phenazine methosulfate, an electron-coupling reagent. In brief, the cells in each well were washed with phosphate-buffered saline (PBS) and then treated with 100 μl of Aqueous One Solution reagent for 30 min. Then, the absorbance at 492 nm was measured using a microplate reader (Apollo LB 9110; Berthold Technologies GmbH, Germany). The percentage of viable cells relative to that of the untreated controls was estimated. The viability assay was repeated three times.

### Evaluation of Intracellular ROS Production

Intracellular ROS production was measured using the 2',7'-dichlorofluorescein diacetate (DCF-DA) assay. In brief, HaCaT cells were first starved for 4 h and then pretreated with different concentrations of MMPP (5 and 10 μg/ml) for 1 h. N-acetyl-L-cysteine (NAC; Sigma), ROS scavenger, was used as a positive control. After 30 min of TNF-α/IFN-γ stimulation, the cells were treated with 10 μM DCF-DA (Sigma, USA) for 45 min, after which the intracellular ROS level was measured. A VICTOR X3 microplate reader (PerkinElmer, USA) was used for the fluorometric analysis. In addition to fluorometry, DCF-DA-treated cells were examined using confocal fluorescence microscopy (EVOSTM M7000 Imaging System; Thermo Fisher Scientific Inc., USA) and a FACSCalibur flow cytometer (BD Biosciences, USA).

### RNA isolation and RT-qPCR

HaCaT cells (3 × 10^5^ cells/ml) were seeded in 6-well plates and grown for 24 h. Subsequently, the cells were pretreated with different concentrations of MMPP (5 and 10 μg/ml) for 1 h and then stimulated with 10 ng/ml TNF-α/IFN-γ for 6 h or 16 h. The treated cells were collected and lysed using the easy-BLUE Total RNA Extraction Kit (iNtRon Biotechnology, Republic of Korea) according to the manufacturer’s instructions. For the reverse transcription-polymerase chain reaction (RT-PCR), 1 μg of RNA was reverse transcribed to cDNA using oligo (dT) primers, M-MuLV reverse transcriptase (New England Biolabs, USA), and the ProSTAR system (Stratagene, USA). The synthesized cDNA was then amplified and quantitated on the Thermal Cycler Dice Real-Time System (Takara Bio, Japan) using the TB Green Premix Ex Taq (Takara Bio) according to the manufacturer’s instructions. The primers used in this study are listed in Table 1. The results were analyzed using the TaKaRa Dice Real-Time System Single (Takara Bio). The expression levels of all target genes were normalized to that of glyceraldehyde-3-phosphate dehydrogenase (GAPDH), which was used as the housekeeping control gene. The quantitative gene expression values were calculated with the ΔΔCT method using data from independent triplicate experiments.

### Enzyme-Linked Immunosorbent Assay

HaCaT cells (3 × 10^5^ cells/ml) were seeded in 6-well plates, pretreated with MMPP, and then stimulated with TNF-α/IFN-γ for 16 h as described in section 2.5. The cell culture supernatant was then collected for enzyme-linked immunosorbent assay (ELISA) of the secreted IL-6 and CCL5 levels using sandwich ELISA kits (R&D Systems, USA). In brief, 100 μl of each supernatant sample was added to coated 96-well plates and incubated for 2 h. Subsequently, the wells were incubated first with 100 μl of detection antibody for 2 h at ambient temperature, followed by a working dilution of horseradish peroxidase-conjugated streptavidin for 20 min. Tetramethylbenzidine (0.4 g/l, Thermo Fisher Scientific Inc.) was added for color development and the reaction was terminated with NH_2_SO_4_. Finally, the absorbance at 450 nm was measured using a microplate reader (Apollo LB 9110; Berthold Technologies GmbH).

### Immunoblot Analysis

HaCaT cells (3 × 10^5^ cells/ml) were seeded in 60 mm culture dishes and pretreated with 5 or 10 μg/ml MMPP for 1 h and then stimulated with 10 ng/ml TNF-α/IFN-γ. The cells were lysed in a 50 mM Tris lysis buffer (pH 7.4), containing 150 mM NaCl, 1% NP40, 0.1% sodium dodecyl sulfate, 0.25% sodium deoxycholate, 1 mM ethylenediaminetetraacetic acid, 1 mM ethylene glycol tetraacetic acid, 1 mm orthovanadate, 10 μg/ml aprotinin, and 0.4 mM phenylmethylsulfonyl fluoride at 4°C for 30 min. After determination of the concentration of total proteins isolated from the harvested cells, equal amounts were separated using sodium dodecyl sulfate-polyacrylamide gel electrophoresis (SDS-PAGE). The separated protein bands were then transferred to a polyvinylidene difluoride (PVDF) membrane. Specific primary antibodies against phosphorylated extracellular signal-regulated kinase (p-ERK) (#9101S; Signaling Technology, USA), phosphorylated c-Jun N-terminal kinase (p-JNK) (#9251S; Cell Signaling Technology), phosphorylated p38 (p-p38) (sc-17852-R; Santa Cruz Biotechnology, USA), and GAPDH (#sc-477244; Santa Cruz Biotechnology) were used to detect the target proteins.

### Luciferase Assay

HaCaT cells (2 × 10^5^ cells/ml) were transfected overnight with either 0.6 μg of the firefly luciferase-expressing NF-κB plasmid vector (Promega) or 0.4 μg of the *Renilla* luciferase-expressing pRL-null control vector, using the OmicsFect DNA transfection reagent (Omics Biotechnology Co., Taiwan). Thereafter, the transfected cells were pretreated with MMPP for 1 h and then stimulated with TNF-α/IFN-γ for 16 h prior to lysis. The promoter activities were measured using the Dual-Luciferase Reporter Assay System (Promega). The firefly luciferase activity in each test sample was measured using a VICTOR X3 plate reader (PerkinElmer Inc., USA) and normalized to that of the control *Renilla* luciferase.

### Cell Fractionation

HaCaT cells (3 × 10^5^ cells/ml) were seeded in 60 mm culture dishes and pretreated with 5 or 10 μg/ml MMPP for 1 h and then stimulated with 10 ng/mL TNF-α/IFN-γ for 2 h. The cells were then harvested and fractionated using NE-PER nuclear and cytoplasmic extraction reagents (Thermo Fisher Scientific Inc.) according to the manufacturer’s instructions. Equal amounts of protein (15 μg) from these fractions were separated using SDS-PAGE and the protein bands were transferred to PVDF membranes. Primary antibodies against c-Jun (#sc-45; Santa Cruz Biotechnology), c-FOS (#sc-52; Santa Cruz Biotechnology), p50 (#sc-8414; Santa Cruz Biotechnology), p65 (#4764; Cell Signaling Technology), and STAT1 (#9172; Cell Signaling Technology) were used for both the cytosolic and nuclear fractions. Poly-ADP ribose polymerase (PARP) (#9542S; Cell Signaling Technology) and GAPDH (#sc-477244; Santa Cruz Biotechnology) were used as nuclear and cytosolic protein markers, respectively.

### Molecular Docking Studies

Docking studies of MMPP in complex with IKKα and IKKβ were performed using PyRx v0.9.2 docking software together with AutoDock Vina v1.2.0. Three-dimensional structures of IKKα (PDB code: 5ebz) and IKKβ (PDB code: 4kik) were used in the docking. Starting with the co-crystallized complexes, a monomer chain was separated from the IKKα and IKKβ complexes, respectively, using PyMOL. The grid box was centered on the protein, with the size adjusted to include the whole protein. Molecular graphics of the best binding model were generated using Biovia Discovery Studio Visualizer v21.1.0.

### Immunofluorescence Staining

HaCaT cells (1 × 10^5^ cells/ml) were seeded in 8-well cell culture slides and pretreated with 5 or 10 μg/ml MMPP for 1 h and then stimulated with 10 ng/ml TNF-α/IFN-γ for 2 h. Subsequently, 4% paraformaldehyde and 100%methanol were used to fix and permeabilize the cells, respectively. Bovine serum albumin (1%) in PBS was used to block the nonspecific binding of antibodies. Then, the cells were incubated overnight at 4°C either a rabbit monoclonal primary antibody against NF-κB p65 (1:200 dilutions) or a mouse monoclonal primary antibody against NF-κB p50 (1:200 dilutions). This was followed by incubation with a FITC-labeled goat anti-rabbit IgG secondary antibody (for p65) (Merck Milipore, Germany) or a Cy3-labeled goat anti-mouse IgG secondary antibody (for p50) (Merck Milipore) (1:400 dilution) for 1 h, respectively. Finally, the cells were stained with 4,6-diamidino-2-phenylindole (1:1000 dilution) (Sigma) for 10 sec at ambient temperature. A confocal fluorescence microscope (EVOSTM M7000 Imaging System; Thermo Fisher Scientific Inc.) with a 100× objective was used to obtain fluorescence images.

### Statistical Analysis

The data obtained from three or more independent experiments are presented as the use mean ± SD. One-way ANOVA and Tukey’s honestly significant difference tests were performed using GraphPad Prism 9 software (GraphPad Software Inc., USA). Differences with a *p*-value of less than 0.05 were considered to be statistically significant.

## Results

### Chemical Structure of MMPP and Its Cytotoxic Effects in HaCaT Cells in the Presence or Absence of TNF-α/IFN-γ

**Fig. 1:**
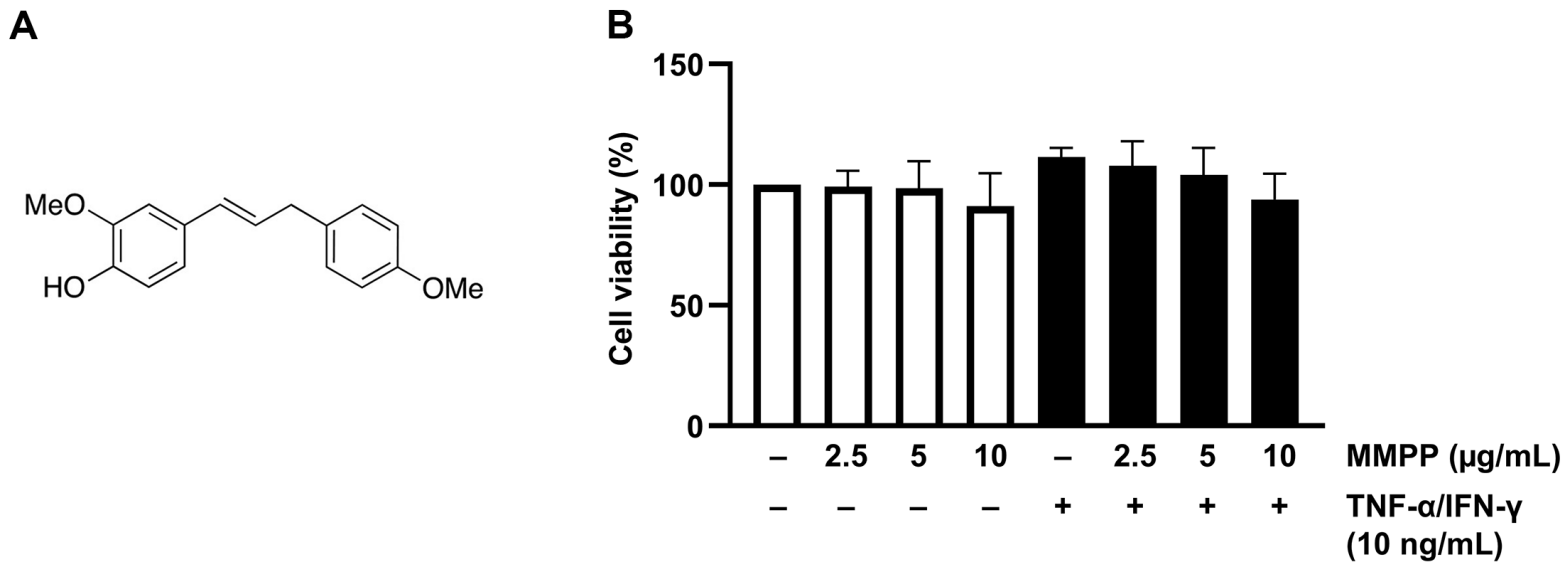
Chemical structure of MMPP and its toxic effect against TNF-α/IFN-γ-stimulated HaCaT cells. (**A**) Chemical structure of MMPP. (**B**) MTS assay results of the effect of MMPP on the viability of TNF-α/IFN-γ-stimulated HaCaT cells. The results represent the use mean ± SD of three experiments.

The chemical structure of MMPP is shown in Fig. 1A. MMPP, up to 10 μg/ml, was tested for cytotoxicity against HaCaT cells in the absence or presence of 10 ng/ml TNF-α/IFN-γ. The cell viability of the HaCaT cells with MMPP was determined. According to the results of the MTS assay, no significant cytotoxicity was observed at any of the MMPP concentrations tested (Fig. 1B).

### Modulatory Effect of MMPP on ROS Levels in TNF-α/IFN-γ-Stimulated HaCaT Cells

**Fig. 2:**
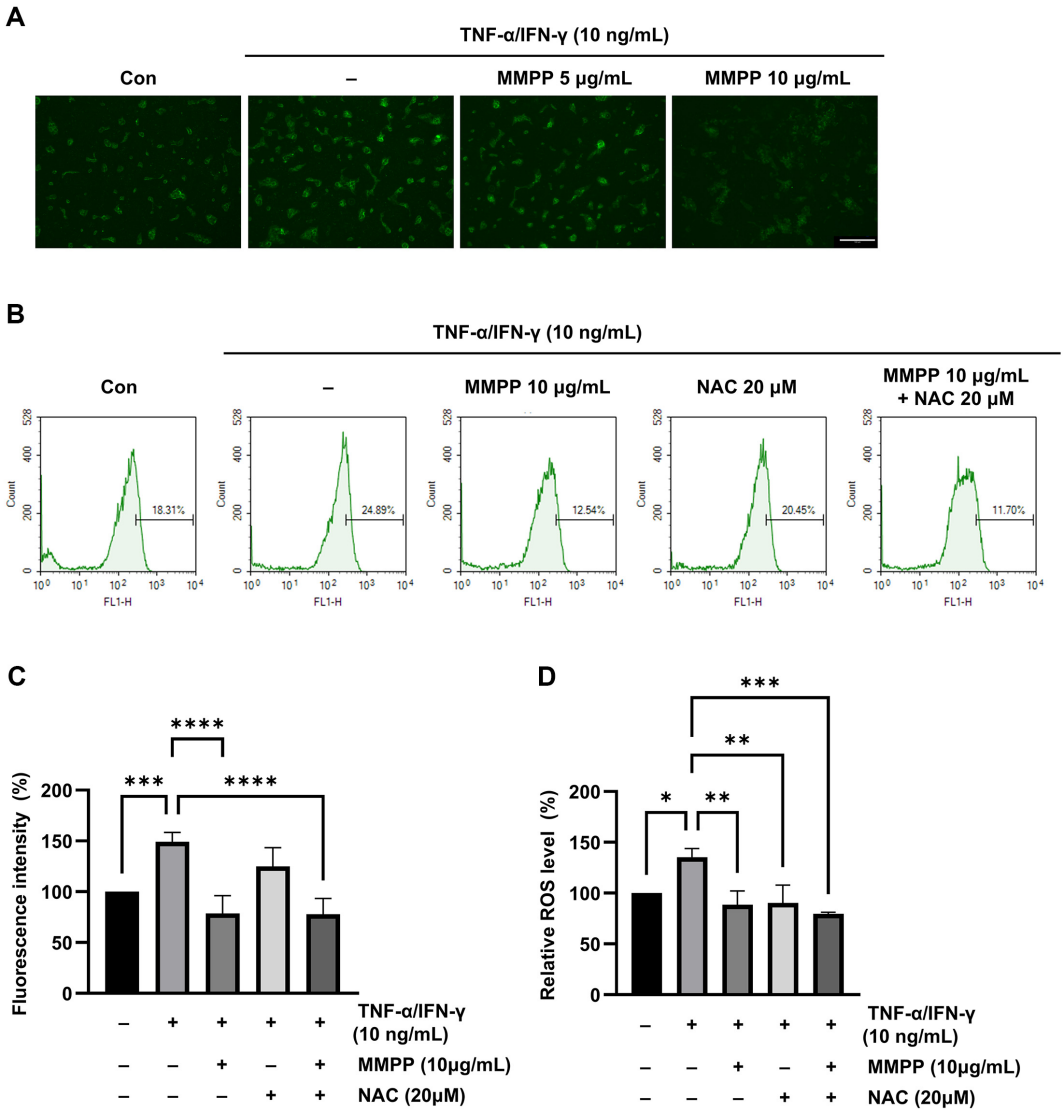
MMPP suppression of intracellular ROS production in TNF-α/IFN-γ-stimulated HaCaT cells. (**A**) Intracellular levels of generated ROS were analyzed using fluorescence microscopy (magnification ×10; scale bar = 275 μm), (**B, C**) flow cytometry, and (**D**) DCF-DA assay. The results shown represent data from three independent experiments (*n* = 3), and values are indicated as the means ± SD. *p < 0.05, **p <0.01, ***p <0.001, ****p <0.0001 by one-way ANOVA.

To investigate whether MMPP can modulate ROS generation in TNF-α/IFN-γ-stimulated HaCaT cells, the keratinocytes were treated with the MMPP or NAC for 1 h and then stimulated with TNF-α/IFN-γ for 30 min [20]. DCF-DA fluorescence imaging showed that TNF-α/IFN-γ stimulation had increased the intracellular ROS levels, as indicated by the high fluorescence intensity compared with that of the non-stimulated control group. However, treatment with MMPP resulted in a dose-dependent decrease in the fluorescence intensity, indicating a reduction in intracellular ROS levels (Fig. 2A). Flow cytometric analysis of the DCF-DA-stained HaCaT cells verified both the increase in intracellular ROS levels after TNF-α/IFN-γ stimulation and the attenuation of this effect by MMPP treatment (Fig. 2B and 2C). Additionally, the microplate reader measurements further confirmed that the intracellular ROS levels were significantly increased by TNF-α/IFN-γ stimulation but reduced in a significant manner by treatment with MMPP or NAC (Fig. 2D). As a positive control, cells were pretreated with 20 μM of NAC and then stimulated in the same manner as for the other cell groups. Moreover, there was no synergistic effect between MMPP and NAC. These results support the conclusion that MMPP suppresses ROS production, similar to NAC.

### Effect of MMPP on the TNF-α/IFN-γ-Induced Downregulation of Antioxidant Genes in HaCaT Cells

**Fig. 3:**
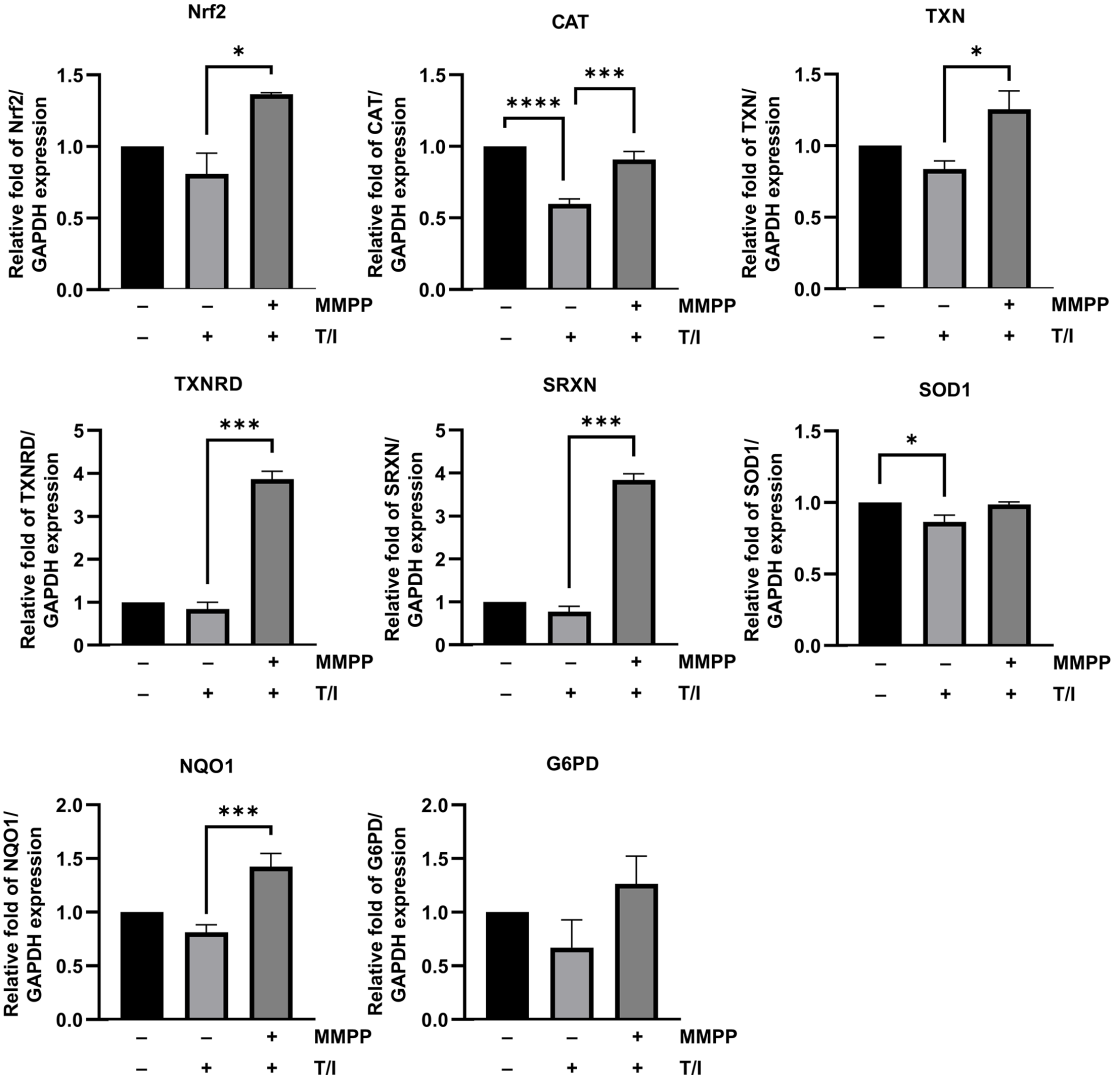
Effects of MMPP on the expression of ROS scavenger and antioxidant genes in TNF-α/IFN-γ-stimulated HaCaT cells. The cells were pretreated with MMPP for 1 h and then stimulated with 10 ng/ml TNF-α/IFN-γ (T/I) for 6 h. The mRNA expression levels of ROS scavenger genes were determined using the RT-qPCR assay. Data are from three independent experiments and reported as the use mean ± SD (*n* = 3). *p <0.05, ***p <0.001, ****p <0.0001 by one-way ANOVA.

Cells possess antioxidant networks that scavenge excess ROS. The balance between ROS production and scavenging is critical for maintaining cellular homeostasis [21]. We assessed whether MMPP could recover the TNF-α/IFN-γ-induced decrease in expression of genes encoding the antioxidants responsible for scavenging ROS. The mRNA levels of the genes encoding Nrf2 and Nrf2 target genes, including CAT, thioredoxin (TXN), thioredoxin reductase (TXNRD), sulfiredoxin (SRXN), SOD1, glucose-6-phosphate dehydrogenase (G6PD), and NQO1 were decreased upon stimulation with TNF-α/IFN-γ, but pretreatment with MMPP restored the expression of all these genes (Fig. 3). These results suggest that MMPP enhances expression of Nrf2 related genes involved in antioxidant and redox regulation.

### Inhibitory Effects of MMPP against TNF-α/IFN-γ-Induced Proinflammatory Cytokine and Chemokine Expression in HaCaT Cells

**Fig. 4:**
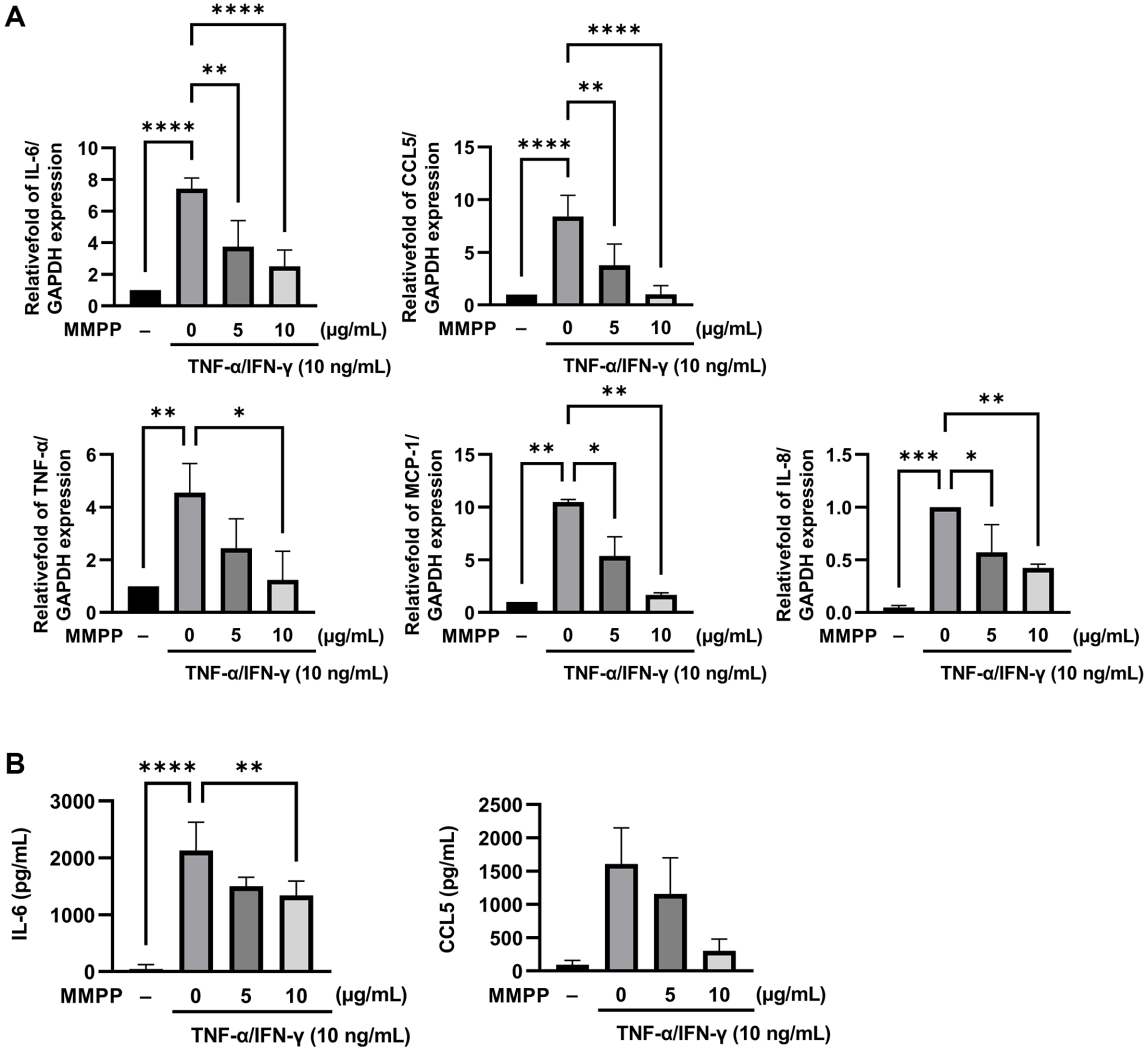
Inhibitory effects of MMPP on the TNF-α/IFN-γ-induced expression of proinflammatory cytokines and chemokines in HaCaT cells. The cells were pretreated with MMPP for 1h and then stimulated with 10 ng/ml TNF-α/IFN-γ for 16h. (**A**) RT-qPCR quantitation of the mRNA expression levels of proinflammatory cytokines and chemokines. (**B**) ELISA results of the levels of IL-6 and CCL5 secreted into the culture medium. Data are from three independent experiments and reported as the use mean ± SD (*n* = 3). *p < 0.05, **p <0.01, ****p <0.0001 by one-way ANOVA.

To investigate whether MMPP affects the mRNA levels of TNF-α/IFN-γ-induced cytokines and chemokines, HaCaT cells were treated with the MMPP for 1 h and then stimulated with TNF-α/IFN-γ for 16 h. RT-qPCR quantitation showed that MMPP inhibited TNF-α/IFN-γ-induced IL-6, CCL5, TNF-α, MCP-1, and IL-8 mRNA expression in a dose-dependent manner (Fig. 4A). Among these genes, IL-6 and CCL5 were more downregulated than the other genes. Therefore, the IL-6 and CCL5 protein secreted into the HaCaT cell culture supernatant were detected using ELISA, which verified their levels were significantly decreased by MMPP (Fig. 4B). These results suggest that MMPP can reduce the mRNA and protein levels of cytokines and chemokines associated with the skin inflammatory response.

### Binding of MMPP to the ATP-Binding Pocket of IKKα/β

**Fig. 5:**
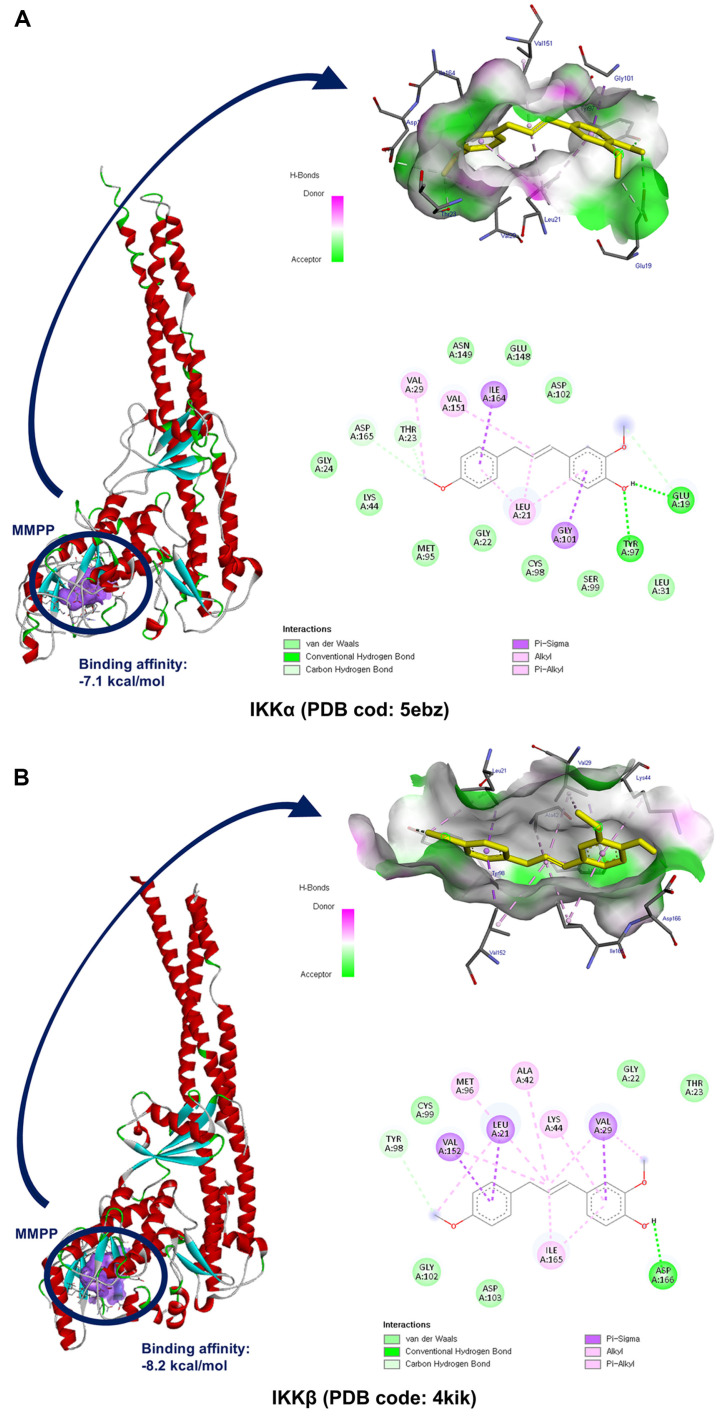
Molecular docking studies of the interaction between MMPP and IKKα/β. MMPP bonded directly to the ATP-binding sites of IKKα and IKKβ with a strong binding affinity (–7.1 and –8.2 kcal/mol, respectively). (**A**) Docking complex of MMPP (CID:122517441; yellow) with IKKα (PDB code: 5ebz). (**B**) Docking complex of MMPP with IKKβ (PDB code: 4kik).

IKKα/β is a critical upstream kinase that activates IκBα and NF-κB. Since NF-κB signaling plays a central role in inflammatory responses, we hypothesized that MMPP may modulate IKKα/β activity by binding. To demonstrate this, we performed an in silico molecular docking experiment to predict the possible binding modes of MMPP to IKKα and IKKβ. Among the protein–ligand complexes generated, the ones with the lowest binding energy were selected and analyzed. The analysis revealed that MMPP bonded directly to the ATP-binding sites of IKKα and IKKβ with strong binding affinities (–7.1 and –8.2 kcal/mol, respectively) (Fig. 5A and 5B). These results indicate that MMPP can bind directly to IKKα/β, affecting downstream signaling pathway.

### Effects of MMPP on the Phosphorylation of IKKα/β, IκBα, and MAPK in TNF-α/IFN-γ-Stimulated HaCaT Cells

**Fig. 6:**
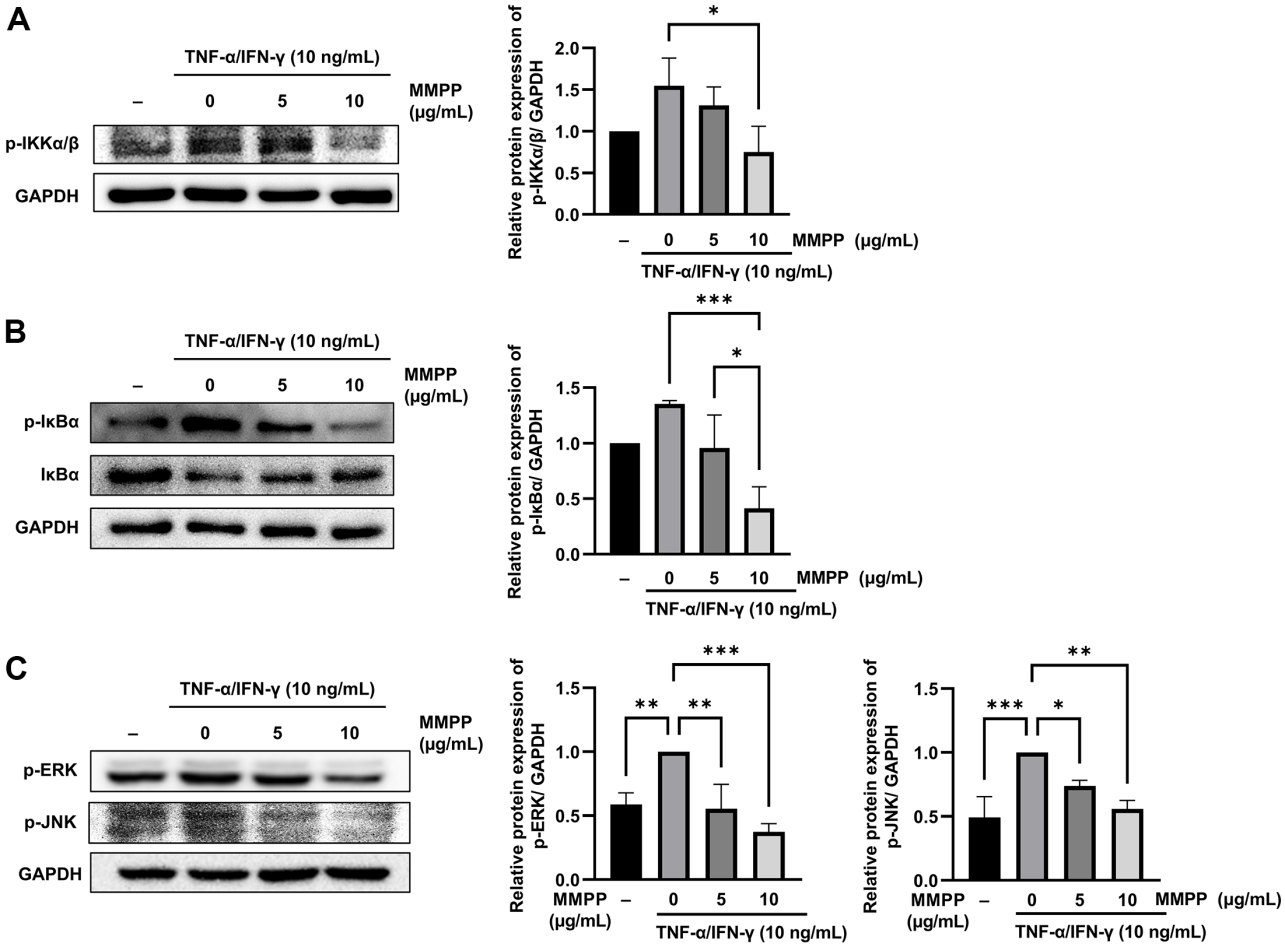
Effects of MMPP on the phosphorylation of IKKα/β, IκBα, and MAPK in TNF-α/IFN-γ-stimulated HaCaT cells. (**A**) Immunoblot of p-IKKα/β levels. HaCaT cells were pretreated with 5 or 10 μg/ml MMPP for 1 h and then stimulated with 10 ng/ml TNF-α/IFN-γ for 15 min. (**B**) Immunoblot of the p-IκBα levels. HaCaT cells were pretreated with 5 or 10 μg/ml MMPP for 1 h and then stimulated with 10 ng/ml TNF-α/IFN-γ for 45 min. (**C**) Immunoblot of the p-ERK and p-JNK levels. HaCaT cells were pretreated with MMPP for 1 h and then stimulated with 10 ng/ml TNF-α/IFN-γ for 1 h. All results represent one of three independent experiments. The relative levels of p-ERK were calculated using ImageJ software. The band intensities were normalized to that of GAPDH and are presented as a fold change. Data represent the use mean ± SD of three experiments. *p < 0.05, **p < 0.01, ***p < 0.001 by one-way ANOVA.

To validate the molecular docking between MMPP and IKKα/β, we examined the inhibitory effect of the MMPP on the downstream IKKα/β signaling pathway, in which IκBα is a key regulator of NF-κB activation [22]. To examine the effects of MMPP on IKKα/β signaling, immunoblot analysis was conducted to assess the phosphorylation status of IKKα/β in the TNF-α/IFN-γ-stimulated HaCaT cells. The results showed that MMPP decreased the levels of p-IKKα/β (Fig. 6A). Because translocated NF-κB can be indirectly measured by quantitating the amount of p-IκBα detached from the NF-κB–IκBα complex, we also quantitated the p-IκBα levels using immunoblotting. The cells were pre-treated with 10 μg/ml of MMPP for 1 h before stimulation with 10 ng/ml TNF-α/IFN-γ for 45 min, and the results showed that MMPP decreased the p-IκBα levels significantly in a dose-dependent manner in the TNF-α/IFN-γ-stimulated cells (Fig. 6B). Additionally, the MAPK pathway plays an important role in various intracellular signaling pathways, including inflammation, through the induction of cytokine and chemokine production. The MAPK family includes ERK, JNK, and p38. Therefore, the effects of MMPP on the TNF-α/IFN-γ-induced phosphorylation of ERK, JNK, and p38 were evaluated. According to the immunoblot results, MMPP reduced the p-ERK and p-JNK levels (Fig. 6C). These findings indicate that TNF-α/IFN-γ enhances the phosphorylation of ERK and JNK, whereas MMPP inhibits the ERK/JNK signaling pathway.

### Effects of MMPP on the Activities of Transcription Factors in TNF-α/IFN-γ-Stimulated HaCaT Cells

**Fig. 7:**
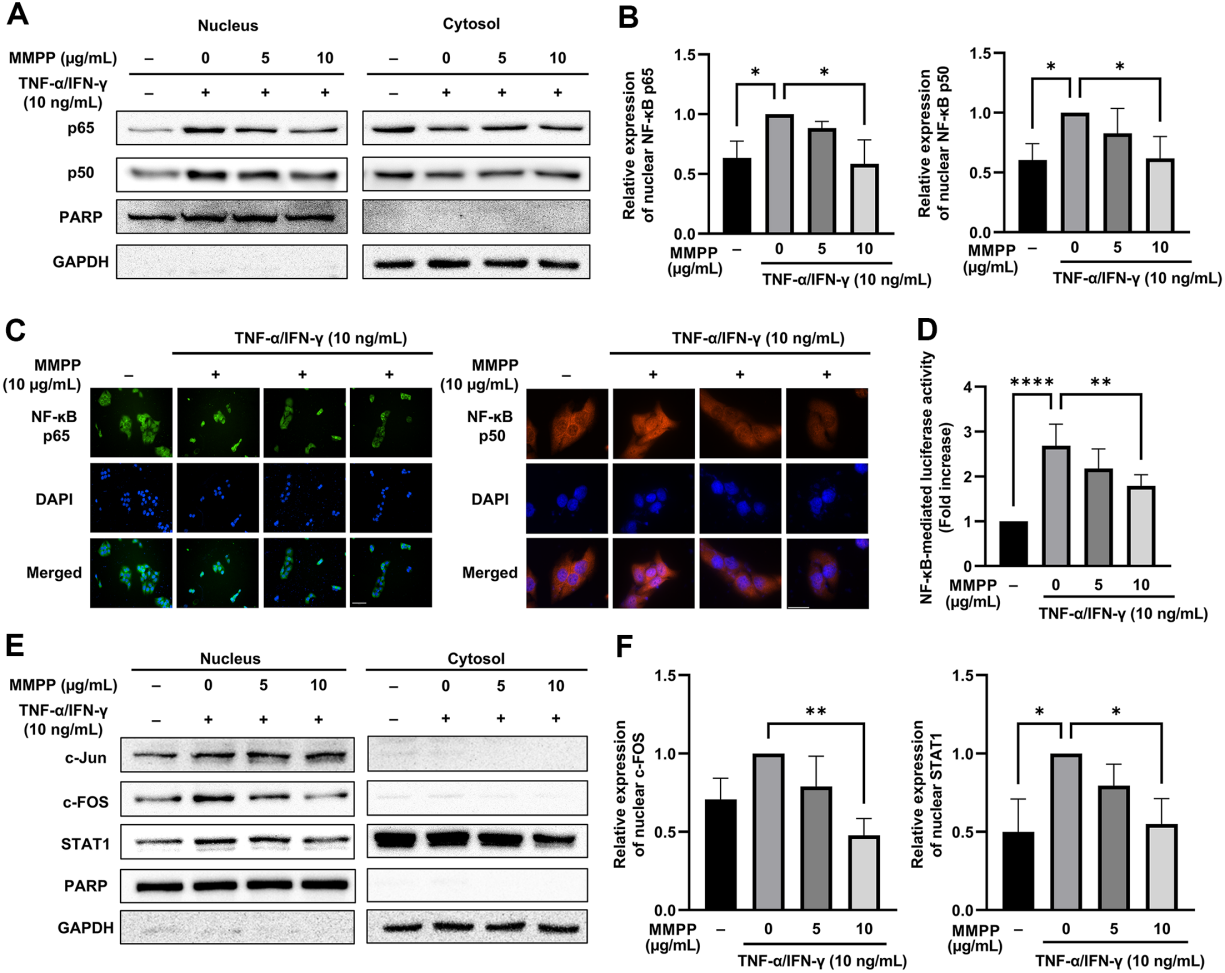
Effects of MMPP on the activities of transcription factors in TNF-α/IFN-γ-stimulated HaCaT cells. (**A**) Immunoblots of the p65 and p50 transcription factors in the nuclear and cytosolic fractions extracted via cell fractionation. (**B**) Relative levels of nuclear p65 and p50, calculated using ImageJ software. The band intensities were normalized to that of PARP and are presented as a fold change. (**C**) Immunofluorescence staining for the cellular localization of p65 and p50. (**D**) Luciferase assay results of the inhibitory effect of MMPP on TNF-α/IFN-γ-induced NF-κB promoter activity. (**E**) Immunoblots of the c-Jun, c-FOS, STAT1 transcription factors in the nuclear and cytosolic fractions extracted via cell fractionation. (**F**) Relative levels of nuclear c-FOS and STAT1, calculated using ImageJ software. The band intensities were normalized to that of the nuclear marker PARP and are presented as a fold change. Data represent the use mean ± SD of three experiments. *p < 0.05, **p < 0.01, ***p < 0.001, ****p <0.0001 by one-way ANOVA.

Phosphorylation and activation of MAPKs trigger the nuclear translocation of transcription factors, which subsequently induce the expression of genes involved in inflammation. Among these transcription factors, NF-κB and AP-1 are particularly notable. Additionally, IFN-γ signaling is known to activate STAT1, which directly induces the expression of immune effector genes, including those encoding chemokines and cytokines [22]. Cellular fractionation was performed to determine the transcription factors mediating the anti-inflammatory activity of MMPP. According to the immunoblot results, MMPP inhibited the nuclear translocation of the NF-κB subunits p65 and p50 (Fig. 7A and 7B). These findings were verified by the immunofluorescence staining results (Fig. 7C). The NF-κB luciferase assay further confirmed that MMPP inhibited the promoter activity of NF-κB induced by TNF-α/IFN-γ (Fig. 7D). Additionally, the nuclear translocation of AP-1 (c-FOS) and STAT1 was also suppressed by MMPP (Fig. 7E and 7F).

### Effects of an NF-κB Inhibitor and MMPP on IL-6 and CCL5 Expression in TNF-α/IFN-γ-Stimulated HaCaT Cells

**Fig. 8:**
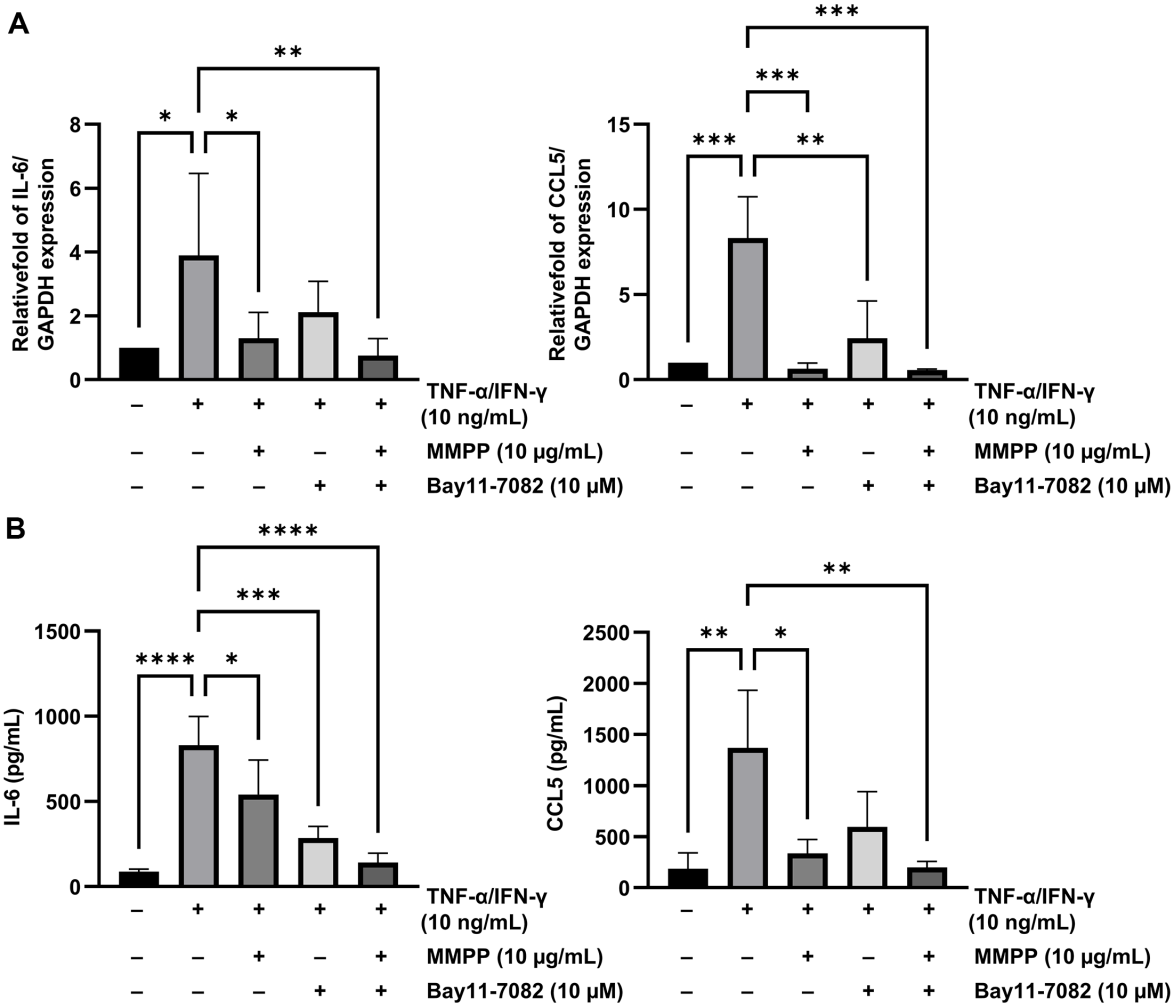
Effects of Bay11-7082 (an NF-κB inhibitor) and MMPP on IL-6 and CCL5 expression in TNF-α/IFN- γ-stimulated HaCaT cells. (**A**) IL-6 and CCL5 mRNA expression levels in HaCaT cells. (**B**) Levels of IL-6 and CCL5 secreted into the culture media of the various HaCaT cell groups. Data represent the use mean ± SD of three experiments. *p < 0.05, **p < 0.01, ***p < 0.001, ****p <0.0001 by one-way ANOVA.

Bay11-7082, an NF-κB inhibitor, inhibits the IκBα degradation and MAPK pathways. Therefore, it was used to investigate whether NF-κB mediates the expression of IL-6 and CCL5 protein. Bay11-7082 and MMPP individually decreased the mRNA expression of IL-6 and CCL5 (Fig. 8A). Moreover, MMPP significantly inhibited TNF-α/IFN-induced IL-6 and CCL5 production regardless of the presence or absence of Bay11-7082 (Fig. 8B). Therefore, MMPP seems to act similarly to the NF-κB inhibitor.

## Discussion

In this study, we revealed that MMPP attenuates inflammation in TNF-α/IFN-γ-stimulated human keratinocyte HaCaT cells. Keratinocytes are essential for maintaining skin immune homeostasis, any disturbances in their function are closely linked to the pathogenesis of atopic dermatitis as well as other inflammatory skin disorders [23]. They contribute to inflammatory skin conditions by releasing proinflammatory cytokines and chemokines, including TNF-α, IL-1β, IL-6, and IL-8 [24]. Specifically, the inflammatory chemokine CCL5/RANTES, a member of the C-C motif chemokine ligand family, is notable for directing mast cells to sites of inflammation [25]. Its expression is elevated in atopic dermatitis lesions [26], while reducing its activity has shown therapeutic benefit in experimental skin inflammation models [27]. Activated keratinocytes also produce Th2-associated chemokines that promote immune cell infiltration into inflamed skin tissue, thereby mediating inflammatory responses in skin diseases [28, 29]. Those chemokines activate and induce antigen-presenting cells to produce inflammatory cytokines such as IL-1 and IL-6. Therefore, modulating inflammatory cytokines and chemokines is an important therapeutic approach for alleviating skin inflammation.

ROS are critical mediators of inflammation, as excessive ROS production can damage skin tissue and promote inflammatory cell infiltration [30, 31]. To counteract this, the NRF2 signaling pathway is activated, which protects against oxidation and inflammation by upregulating the expression of ROS scavengers and antioxidative enzymes and inhibiting ROS production, thereby preventing cellular damage [32, 33].

The TNF-α/IFN-γ combination is known to activate several intracellular pathways related to inflammatory skin diseases [34]. Also, many studies show that TNF-α/IFN-γ treatment increases ROS production and results in the increased cytokine and chemokine production in keratinocytes [35]. In this study, we found that the TNF-α/IFN-γ-induced increase in ROS level was reduced by MMPP. Furthermore, MMPP decreased the TNF-α/IFN-γ-induced increases in expression of the NRF2-encoding genes, which resulted in the upregulated expression of ROS scavengers and antioxidative enzymes, such as CAT, TXN, TXNRD, SRXN, SOD1, G6PD, and NQO1. Moreover, MMPP treatment notably reduced the production of IL-6, IL-8, TNF-α, MCP-1, and CCL5 in HaCaT cells, which were significantly increased by TNF-α/IFN-γ. Taken together, MMPP enhances the cellular antioxidant defense mechanisms and reduces the production of proinflammatory cytokines and chemokines, thereby protecting cells from oxidative stress and inflammatory responses.

A major finding of this study is that MMPP directly targets the IKK complex, inhibiting IKKα/β phosphorylation and preventing IκBα degradation. This leads to reduced NF-κB nuclear translocation and downstream suppression of proinflammatory cytokines and chemokines, such as TNF-α, IL-1, IL-6, and IL-8 [36, 37]. Upon stimulation of the cells, the IKK complex becomes activated and phosphorylates IκBα, which are dissociated from the p65 and p50 NF-κB subunits and allows NF-κB activation. The released NF-κB then translocates to the nucleus and binds to the NF-κB-binding sites in the promoter region of the target genes. Consequently, IκBα phosphorylation by IKKα/β serves as a critical step in initiation of NF-κB signaling [38, 39]. In the molecular docking study, we demonstrated that MMPP fits within the catalytic pocket of the IKK complex and forms stabilizing interactions with key residues in both IKKα and IKKβ, supporting direct target engagement. Consistently, immunoblot analyses revealed that MMPP inhibited the phosphorylation of IKKα/β and IκBα, thereby preventing NF-κB nuclear translocation. Together, these findings support the conclusion that MMPP binds to IKK and functionally blocks the canonical IKK–IκBα–NF-κB axis.

In addition, the MAPK family proteins ERK, JNK, and p38 are speculated to play different roles in chronic inflammatory diseases and skin homeostasis [38, 39]. These MAPK cascades alter the phosphorylation and activation of transcription factors, leading to the induction of target genes involved in inflammatory responses within the cytoplasm or nucleus [40]. In this study, we confirmed that MMPP attenuated the phosphorylation of ERK/JNK, indicating its ability to modulate these pathways. MMPP also inhibited the nuclear translocation of AP-1 (c-FOS) and STAT1.

To verify the inhibitory effect of MMPP on the NF-κB signaling pathway, luciferase activity assays were performed with the pGL3-NF-κB‐Luc plasmid. The relative luciferase activity was markedly lowered in the MMPP-pretreated HaCaT cells, with the inhibitory effect occurring in a dose‐dependent manner. Additionally, we examined whether IL-6 and CCL5 upregulation is mediated by NF-κB. The NF-κB inhibitor Bay11-7082 significantly suppressed the mRNA expression and secreted protein levels of IL-6 and CCL5. The results of this analysis suggest that MMPP inhibits the transcription of NF-κB and thereby the mRNA expression of cytokine and chemokine genes similarly to the effects of the NF-κB inhibitors [41].

Taken together, this study demonstrates that MMPP exerts potent anti-inflammatory effects in TNF-α/IFN-γ-stimulated HaCaT cells, primarily through direct binding to IKKα/β and subsequent inhibition of the canonical IκBα–NF-κB pathway. In addition, MMPP suppresses the production of proinflammatory cytokines and chemokines by downregulating ERK/JNK, AP-1, and STAT1 activation. Rather than acting on a single pathway, MMPP simultaneously attenuates multiple signaling axes, underscoring its potential as a broad-spectrum inhibitor of keratinocyte-mediated inflammatory responses. Collectively, these properties highlight MMPP as a promising therapeutic candidate for inflammatory skin disorders, in which both oxidative stress and cytokine-driven inflammation play central pathogenic roles.

Despite the promising findings, this study has the following limitation. All experiments were performed using an *in vitro* keratinocyte model, which may not fully reflect the complexity of skin tissue and immune cell interactions *in vivo*. Future studies employing animal models and clinical samples will be necessary to validate the anti-inflammatory and antioxidant effects of MMPP under more physiologically relevant conditions.

## Figures and Tables

**Table 1:** Primer sequences for the cytokine and antioxidant genes.